# Prognostic value of serum heart-type fatty acid-binding protein in patients with sepsis: A protocol for systematic review and meta-analysis

**DOI:** 10.1097/MD.0000000000024715

**Published:** 2021-02-12

**Authors:** Dagang Yang, Guojuan Liu, Shuang Guo, Huibang Ren, Hongnan Lu, Lihua Zhou, Longtang Bao

**Affiliations:** a Department of Critical Care Medicine, Affiliated Hospital of Inner Mongolia Medical University; b Department of Critical Care Medicine, Inner Mongolia Autonomous Region People's Hospital; c Department of Surgical Anaesthesia, Traditional Chinese Medicine Hospital of Inner Mongolia Autonomous Region; d Emergency ICU, Qinghai Provincial People's Hospital; e Emergency Department, First Affiliated Hospital of Jiamusi University; f Daytime Ward, Affiliated Hospital of Inner Mongolia Medical University, China.

**Keywords:** heart-type fatty acid–binding protein, mortality, sepsis, systematic review

## Abstract

**Background:**

Sepsis is commonly acute and critical illness with high morbidity and high mortality, and requires timely diagnosis and treatment. Septic patients had elevated serum H-FABP levels, which may correlate with disease severity and mortality. However, previous studies showed that the association between H-FABP and mortality during the sepsis remains unclear. Thus, we performed a study to analyze this relationship.

**Methods:**

The electronic databases such as Cochrane Library, PubMed, Embase, Web of Science, Cochrane Clinical Trials Database, Wanfang Database, and China National knowledge Infrastructure (CNKI) were systematically searched to determine the qualified clinical trials. The study language is limited to English or Chinese. The 2 authors used Cochrane Risk of Bias Tool v.2.0 to independently check the quality of papers and extract relevant data. Comprehensive analysis of data extracted in the research using appropriate statistical methods.

**Results:**

Evaluation of the relationship between the prognosis of patients with sepsis and serum H-FABP is the result of this study.

**Conclusion:**

The analysis results of this study can infer that H-FABP may be an independent risk factor for the prognosis of patients with sepsis. It is also helpful for clinical workers to make early evaluation and early treatment of patients with sepsis.

**Ethics and dissemination:**

The conclusions of this meta-analysis study are based on the published evidence. Therefore, moral recognition is unnecessary.

**OSF registration number:**

DOI: 10.17605/ OSF.IO / 2V4HN.(https://osf.io/2v4hn/).

## 1 Introduction

Sepsis is a syndrome caused by infection, which causes abnormal physiological and pathological changes of body, resulting in the release of many inflammatory factors, resulting in damage to variety organ functions; it emphasizes importance of unbalanced response of body to infection, and its potential lethality is far greater than that of simple infection.^[1,2]^ The pathogenesis of sepsis is extremely complex, so the treatment of sepsis in clinic is difficult.^[3]^ Finding out risk factors related to the poor prognosis of patients with sepsis is significant for improving the risk stratification of sepsis and developing new therapeutic targets.^[4]^ Heart-type fatty acid binding protein (H-FABP) exists specifically in cardiomyocytes and has the characteristics of low molecular weight.^[5]^ Its main function is to help cardiomyocytes metabolize fatty acids. Under physiological conditions, the content of plasma central fatty acid binding protein is very low, but when cardiac myocytes are damaged, it can be detected because of its low molecular weight, which can leak through the myocardial cell membrane into the plasma in the early stage of myocardial injury.^[6,7]^ It can be seen that H-FABP has a certain significance in the early diagnosis or persistent injury of myocardial injury in patients with sepsis.^[8,9]^ At present, it has been found that H-FABP is elevated in the serum of patients with sepsis.^[10,11]^ However, no trial has tried to explain the relationship between H-FABP and the prognosis of septic patients, such as ventilator use, vasoactive drug use, 28-day mortality, and so on. Therefore, we conduct a systematic review and meta-analysis of this issue, aiming to comprehensively evaluate the prognostic value of H-FABP in patients with sepsis and provide evidence-based medicine for clinical treatment.

## 2 Methods

### 2.1 Registration

This protocol has been registered on the Open Science Framework (OSF, http://osf.io/) with the registered DOI number is 10.17605/ OSF.IO / 2V4HN.(https://osf.io/2v4hn/). We drafted this protocol based on the “Preferred Reporting Project for Systematic Reviews and Meta-Analysis Protocols” (PRISMA-P).

### 2.2 Eligibility criteria

#### 2.2.1 Type of studies

This research will include random or non-random experimental research, as well as any observational studies to investigate the prognostic significance of H-FABP in patients with sepsis.

#### 2.2.2 Type of participants

Study is included if the participant is ≥18 years of age and is diagnosed with sepsis. In terms of the definition and understanding of sepsis and septic shock, we accept the author's choice.

#### 2.2.3 Type of prognostic factors

The study of H-FABP as a prognostic factor will be included in this protocol, and we will also accept every evaluation of H-FABP provided by the study author. After adjusting for other covariates, we will evaluate the role of H-FABP as prognostic factors. Based on literature search and discussions with clinician reviewers, we formulated the following core adjustment factors: age, severity score (sequential organ failure assessment SORA (SOFA), acute physiology assessment and chronic health assessment II (APACHE II), complications (immunosuppression, cardiopulmonary disease, cancer, digestive system diseases, etc.). We will adjust the above factors based on the new evidence found.

#### 2.2.4 Type of outcomes

##### 2.2.4.1 The main results

The main results will include:

1. 28-day mortality;
2. All due to hospital mortality.

##### 2.2.4.2 Secondary results

Secondary results will include:

1. Length of stay in intensive care unit;
2. Length of stay of survivors;
3. Mechanical ventilation ratio;
4. Use of vasoactive drugs;
5. The proportion of cardiac dysfunction.

### 2.3 Search methods for the identification of studies

#### 2.3.1 Database retrieval

We plan to systematically review and search the literature to identify potential appropriate trials from the electronic database, and systematically search the electronic databases such as the Cochrane Library, PubMed, Embase, Web of Science, Cochrane Clinical Trials Database, Wanfang Database, and China's National knowledge Infrastructure (CNKI) to determine qualified clinical trials. In addition, the language restriction is English or Chinese. The search string for this study includes terms related to population (sepsis), prognostic factors (H-FABP), prognosis (mortality), and prognostic research methods.

#### 2.3.2 Search for other resources

We plan to search for relevant resources and citations from the published references to activate more research materials. In addition, we also plan to try to get the author's response to get more relevant data.

### 2.4 Data collection and analysis

#### 2.4.1 Selection of studies

**Figure 1:**
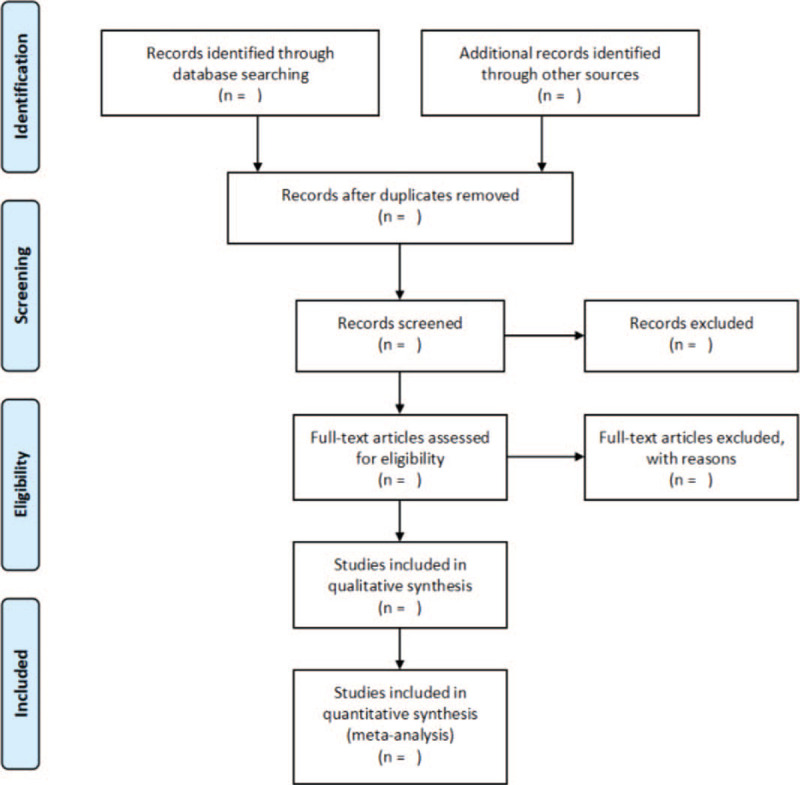
Flow diagram of the literature search. From J Clin Epidemiol. 2009. 6210(10):1009.

For the results of the search, we will select 2 impartial authors to evaluate them: they independently screen the titles, abstracts, and full texts to identify trials that require further evaluation. If there is a disagreement, we will resolve any disagreement through discussion or negotiation with the third author. Figure 1 shows the article screening process.

#### 2.4.2 Data extraction and management

The 2 authors used data excerpt tables to obtain data from 6 areas in each study:

1. The characteristics of the study were published in the year, country, and background.
2. The definition of sepsis used by the author.
3. Participants’ average age, age range, gender, race, details of receiving treatment.
4. Review the results of the study: mortality; measurement type (binary); follow-up period (the first 7 days, the first 28 days and the longest follow-up period provided by the study authors).
5. The results state primary and secondary results, the results collected, and the time point of the report.
6. Pay attention to outcome of the trial and obvious conflicts of interest that must be revealed by the trial author. The third author will participate in the discussion and negotiation of opinion analysis.

#### 2.4.3 Assessment of methodological quality and risk of bias

The 2 authors will use Cochrane Collaborations’ “risk of bias” tool to fairly review the risk of bias identified in this study.^[12]^ The characteristics of the study included:

1. participation in the study;
2. study attrition;
3. measurement of prognostic factors;
4. measurement results;
5. adjustment of other prognostic factors; and
6. statistical analysis and reporting.

In addition, we try to further classify each potential source of bias as “low risk”, “high risk” or “unclear risk”. In addition, the third author will participate in the discussion and negotiation of opinion analysis.

#### 2.4.4 Assessment of heterogeneity

Chi^2^ statistics and *I*^*2*^ test were used to evaluate the heterogeneity. For the statistical result *I*^*2*^ test is greater than 50%, it will be considered that there may be obvious heterogeneity. We choose the fixed effects model to aggregate data by default. For highly heterogeneous data, we will choose a random effects model for data aggregation.^[13,14]^

#### 2.4.5 Assessment of reporting biases

When trials more than 10 cases, we plan to use the funnel chart to detect publication bias.

#### 2.4.6 Data synthesis

Where meta-analysis is used, we default to using fixed-effects models to aggregate results. We will use a random effects model to deal with the heterogeneity of statistical data. We extract association measures (OR, RR, HR) and their standard errors (SE) or confidence intervals (CI) from each study and prognostic assessment. We will convert the relevance metrics to OR and its 95% CI for statistical aggregation appropriately. We plan to set a group of core adjustment coefficients to deal with the situation that the prognostic effect change after a specific factor in the review results which is adjusted.

#### 2.4.7 Sensitivity analysis

If there is enough data, we will conduct a sensitivity check on the research results to ensure their robustness and exclude studies with poor quality or unclear methodological data.

#### 2.4.8 Patient and public participation

The entire research process and the dissemination of research results do not involve patients and the public.

#### 2.4.9 Ethics and communication

The conclusions of this meta-analysis study are based on the published evidence. Therefore, moral recognition is unnecessary. Peer-reviewed publications, conferences, and media dissemination are the main means of dissemination of our research results.

## 3 Discussion

In general, sepsis still has a high morbidity and mortality rate in the rescue and treatment of critically ill patients.^[15]^ Although there has been some progress in the understanding and treatment of sepsis with the development of medical treatment, the prognosis of sepsis is still not optimistic.^[16]^ Some clinical trials have found that serum H-FABP in patients with sepsis is on the rise, but the value of H-FABP in the prognosis of patients with sepsis is currently not systematically reviewed or meta-analyzed. Therefore, we intend to conduct a systematic review and meta-analysis of the prognostic value of H-FABP in patients with sepsis, to determine the independent prognostic risk factors of patients with sepsis, and then provide reference for clinical treatment. The result is that patients with sepsis make use of past and existing clinical studies to lay a foundation for clinical diagnosis and treatment.

## Acknowledgments

The authors thank Rui Xun, Yongxin Yan, Ying Zhang for their support.

## Author contributions

**Conceptualization:** Dagang Yang, Longtang Bao.

**Data curation:** Shuang Guo, Guojuan Liu.

**Formal analysis:** Huibang Ren, Hongnan Lu.

**Funding acquisition:** Longtang Bao.

**Investigation:** Dagang Yang.

**Methodology:** Lihua Zhou.

**Project administration:** Longtang Bao.

**Resources:** Shuang Guo, Guojuan Liu.

**Software:** Huibang Ren, Hongnan Lu.

**Validation:** Lihua Zhou.

**Visualization:** Dagang Yang.

**Writing – original draft:** Dagang Yang.

**Writing – review & editing:** Longtang Bao.

## Abbreviations

Abbreviation: H-FABP = heart-type fatty acid binding protein.
